# Dried *Nostoc commune* exhibits nitrogen-fixing activity using glucose under dark conditions after rehydration

**DOI:** 10.1080/15592324.2022.2059251

**Published:** 2022-04-06

**Authors:** Shingo Hata, Shoji Kishida, Risa Minesono, Tesshu Tamai

**Affiliations:** a Science, Kyoto University, Otsu, Japan; b Agriculture, Ryukoku University, Otsu, Japan; c Takatsuka-cho, Hirakata, Japan; d Agriculture, Kobe University, Otsu, Japan

**Keywords:** Aerobic glucose metabolism, cyanobacteria, energy, nitrogen fixation, photosynthesis, polysaccharide

## Abstract

*Nostoc commune* is an edible cyanobacterium that produces a massive gelatinous polysaccharide matrix around the filamentous cells. The polysaccharides, more than 70% of which comprise glucose, are essential for resistance to environmental stresses. In the present study, we collected naturally growing *N. commune* colonies, dried them for preservation, rehydrated them, and then examined their nitrogen-fixing activity using the acetylene reduction method. As expected, the rehydrated *N. commune* performed nitrogen fixation after illumination with white light. Notably, under dark, aerobic conditions, the rehydrated *N. commune* exhibited nitrogen fixation in the presence of glucose. In contrast, under dark, anaerobic conditions, nitrogen fixation was low. Because the natural habitats of *N. commune* are aerobic but lack carbohydrates, *N. commune* cells may exhibit glucose utilization activity constitutively.

*Nostoc commune* Vauch is a terrestrial cyanobacterium found worldwide that exhibits both photosynthesis and nitrogen fixation.^1^ Typical *N. commune* (var. commune) and its cognate *N. commune* var. flagelliforme (formerly *Nostoc flagelliforme*) form massive sheet-like and thread-like, respectively, gelatinous structures when wet, which become inactive crusts when dry.^1–3^ The filamentous cells in dried colonies survive for more than 100 years without differentiation into akinetes/spores.^4,5^ They exhibit resistance to extreme environmental stresses such as drought,^6^ UV light,^7^ freezing,^8^ and vacuum,^9^ resuming their metabolism rapidly after rehydration. Extracellular polysaccharides play a crucial role in this resistance.^10^ *N. commune* has been traditionally eaten in Asian countries such as China and Japan. Recently, our group demonstrated the usefulness of *N. commune* as an ingredient of buckwheat noodles.^11^ Pharmacological effects of *N. commune* are also gaining interest.^12^ In *N. commune*, nitrogen fixation occurs in heterocysts – specialized cells with thick walls. In the present study, we examined the relationship between nitrogen fixation and the energy source of *N. commune*.

Naturally grown colonies of *N. commune* were collected on the Seta campus of Ryukoku University, Otsu, Shiga, Japan. They were rinsed with tap water to remove soil and then air-dried in a laboratory at room temperature for about 1 week. The dried colonies were stored at 4°C for some months until use. The following experiments were performed using three biological replicates. Dried colonies (0.1 g) were placed in a plastic dish and rehydrated by adding 10 mL distilled water containing 50 µg kanamycin and 0.4 mg cycloheximide (Supplementary Figure S1a and b). The antibiotics were included in the rehydration liquid to prevent contamination by microorganisms. Although kanamycin is generally known to inhibit protein synthesis of prokaryotes, Katoh et al. demonstrated that the low concentration of kanamycin is not toxic for *N. commune*.^13^ Exogenous energy sources, such as glucose and sucrose, were also added to the rehydration liquid. The plastic dishes with rehydrated colonies were illuminated with white fluorescent light (15.5 µmol s^−1^ m^−2^ 14 h day/6.3 µmol s^−1^ m^−2^ 10 h night) at room temperature. To achieve dark, aerobic conditions, each dish containing the colonies was completely shielded from light using aluminum foil. To achieve dark, anaerobic conditions, the light-shielded dishes were placed in a vacuum desiccator, and the air was substituted with nitrogen gas; some pieces of oxygen absorber Ageless (Mitsubishi Gas Chemical, Tokyo) were also placed in the desiccator. After the treatments, the rehydrated colonies were transferred to a 20 mL glass vial and sealed with silicone rubber (Supplementary Figure S1c). Acetylene (1 mL) was added to each vial with the use of a syringe, and the colonies were incubated for 60 min at room temperature. Then, 1 mL of the gas in each vial was subjected to gas–liquid chromatography (Shimadzu GC8A; active alumina column, 1 m long; 100°C), and the conversion from acetylene to ethylene was measured (Supplementary Figure S2).

The *N. commune* colonies swelled soon after rehydration (Supplementary Figure S1a and b) as reported previously,^10,14,15^ but nitrogen fixation was undetectable immediately after rehydration (data not shown). However, under illuminated, aerobic conditions, the nitrogen-fixing activity gradually increased until 96 h and then reached a plateau (Figure 1). These results are consistent with previous findings that rehydration, respiration, photosynthesis, and nitrogen fixation occur in this order.^14,15^ Heterocyst differentiation appears to be relatively slow. That is why nitrogen fixation in heterocysts was resumed slower than photosynthesis that occurs in vegetative cells. Evidently, in this case, the energy source of nitrogen fixation is photosynthesis. Then, we analyzed nitrogen fixation of rehydrated colonies after incubation at room temperature for 96 h.

**Figure 1.**
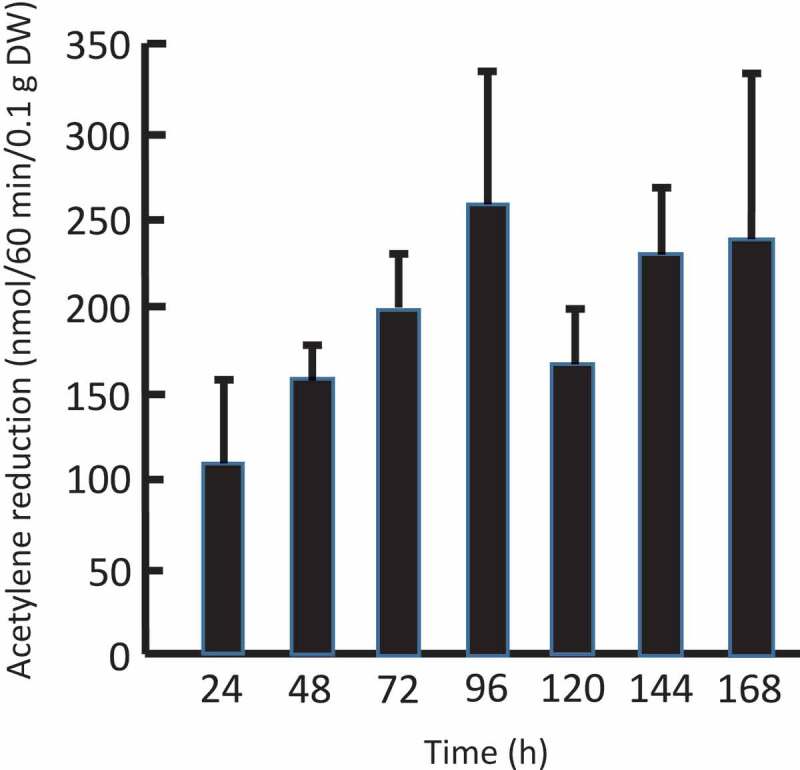
Time course of nitrogen-fixing activity of *Nostoc commune* colonies after rehydration. The experiments were performed under aerobic conditions under white fluorescent light in the absence of exogenous carbohydrates. Results are expressed as mean ± standard deviation (*n* = 3).

Next, we examined nitrogen fixation under dark, aerobic conditions. Colonies rehydrated with only water did not show detectable activity under dark conditions (data not shown). Notably, however, in the presence of 2% (w/v; 111 mM) exogenous glucose, the rehydrated colonies showed one-third of the highest nitrogen-fixing activity observed in illuminated colonies (Figures 1 and 2). The *N. commune* cells seem to have catabolized glucose efficiently and obtained enough energy to perform nitrogen fixation. The presence of sucrose (2% w/v; 58 mM) resulted in lower nitrogen-fixing activity than that in the presence of glucose (Figure 2). The use of pyruvate, 2-oxoglutarate, glycerol, L(+)-arabinose, Na-gluconate, or xylitol (2% w/v each) did not lead to detectable nitrogen-fixing activity (Figure 2 and data not shown). Finally, under dark, anaerobic conditions, rehydrated colonies showed low nitrogen-fixing activity in the presence of glucose but no detectable nitrogen fixation in the presence of sucrose (Figure 2). Obviously, much less energy was obtained under anaerobic conditions than under aerobic conditions. At present, we cannot conclude whether the low activity under dark, anaerobic conditions is due to anaerobic glycolysis by *N. commune* cells or due to aerobic respiration of the cells with residual oxygen. Nonetheless, aerobic glucose metabolism seems to be essential as the energy source for high nitrogen fixation under dark. In addition, we cannot exclude a possibility that anaerobic conditions had inhibited the recovery of nitrogen-fixing activity.

**Figure 2.**
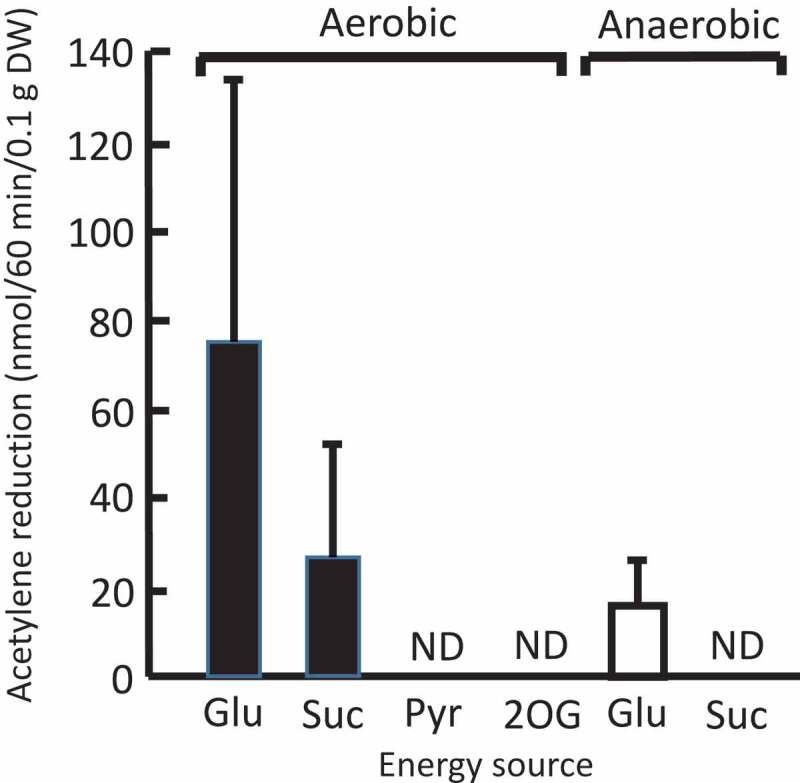
Nitrogen-fixing activity in the dark conditions under aerobic and anaerobic conditions (on the left and right, respectively). The rehydrated colonies of *N. commune* were incubated for 96 h at room temperature in the presence of each energy source, and then their nitrogen fixation was measured. Glu, glucose (2% w/v); Suc, sucrose (2% w/v); Pyr, pyruvate (2% w/v); and 2OG, 2-oxoglutarate (2% w/v). Results are expressed as mean ± standard deviation (*n* = 3). ND, not detectable.

Yu et al. demonstrated that *N. commune* var. flagelliforme showed better growth in a mixotrophic culture containing glucose than under simple photoautotrophic or heterotrophic culture conditions.^16^ The present findings suggest that the typical *N. commune* also has a mixotrophic character. However, in its natural habitats, *N. commune* grows under carbohydrate-poor conditions, for example, on limestone pavements where wet and dry conditions are cyclical.^1^ This is in sharp contrast with the case of another *Nostoc* species and *Anabaena*, symbionts of *Gunnera* and *Azolla*, respectively, in which nitrogen-fixing activity is induced and maintained by a continuous supply of host-derived monosaccharides, especially fructose.^17–19^ Therefore, the following question is pertinent: why did *N. commune* retain the glucose metabolism activity immediately after rehydration? The simplest answer would be that the glucose utilization activity of *N. commune* is constitutive. In this regard, it is noteworthy that 73% (w/w) of the massive extracellular polysaccharide matrix consists of glucose.^20^ When *N. commune* cells are illuminated and energy rich, the intracellular levels of glucose must be high because it is the main material of extracellular polysaccharides. During sudden dark conditions, for example, the constitutive glucose utilization capacity must be advantageous because *N. commune* cells can use the components of the polysaccharide matrix as the energy source for nitrogen fixation.

## Supplementary Material

Supplemental Material
