# Supplementary material for: Dried Nostoc commune exhibits nitrogen-fixing activity using glucose under dark conditions after rehydration
Source: Plant Signal Behav. 2022 Apr 6;17(1):2059251. doi: 10.1080/15592324.2022.2059251 (PMC8993094; doi:10.1080/15592324.2022.2059251)
Supplement: Supplemental Material [file KPSB_A_2059251_SM6494.pptx]

## Slide 1
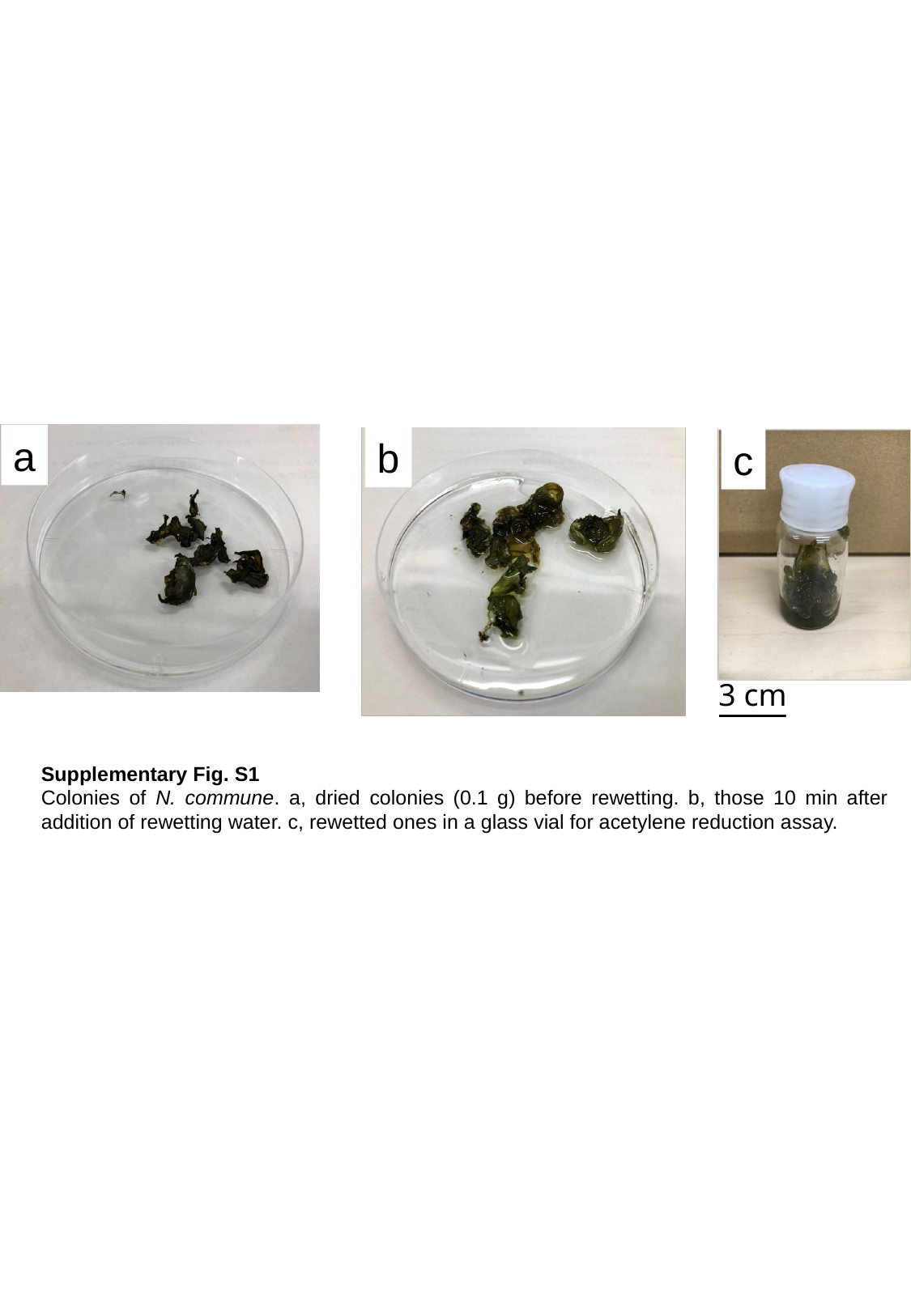

a
b
c
3 cm
Supplementary Fig. S1
Colonies of N. commune. a, dried colonies (0.1 g) before rewetting. b, those 10 min after addition of rewetting water. c, rewetted ones in a glass vial for acetylene reduction assay.

## Slide 2
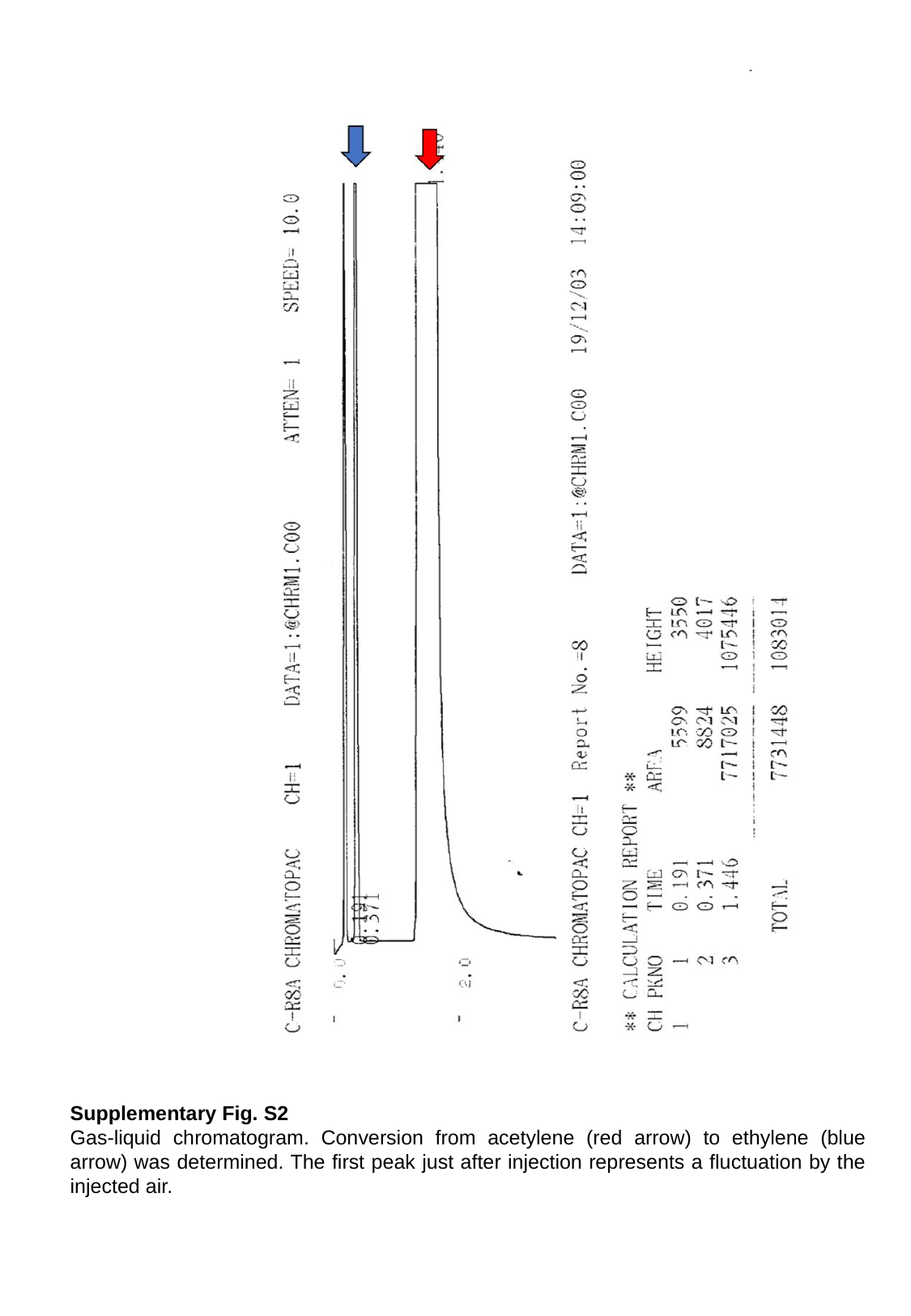

Supplementary Fig. S2
Gas-liquid chromatogram. Conversion from acetylene (red arrow) to ethylene (blue arrow) was determined. The first peak just after injection represents a fluctuation by the injected air.
